# “You can’t swim well if there is a weight dragging you down”: cross-sectional study of intimate partner violence, sexual assault and child abuse prevalence against Australian nurses, midwives and carers

**DOI:** 10.1186/s12889-022-14045-4

**Published:** 2022-09-12

**Authors:** Elizabeth McLindon, Kristin Diemer, Jacqueline Kuruppu, Anneliese Spiteri-Staines, Kelsey Hegarty

**Affiliations:** The University of Melbourne, Melbourne, Australia

**Keywords:** Intimate partner violence, Sexual assault, Child abuse, Nursing, Midwifery, Health professionals, Health services, Cross-sectional survey

## Abstract

**Background:**

Domestic and family violence (DFV), including intimate partner violence (IPV), sexual assault and child abuse are prevalent health and social issues, often precipitating contact with health services. Nurses, midwives and carers are frontline responders to women and children who have experienced violence, with some research suggesting that health professionals themselves may report a higher incidence of IPV in their personal lives compared to the community. This paper reports the largest study of DFV against health professionals to date.

**Method:**

An online descriptive, cross-sectional survey of 10,674 women and 772 men members of the Australian Nursing and Midwifery Federation (ANMF) (Victorian Branch). The primary outcome measures were 12-month and adult lifetime IPV prevalence (Composite Abuse Scale); secondary outcomes included sexual assault and child abuse (Australian Bureau of Statistics Personal Safety Survey) and prevalence of IPV perpetration (bespoke).

**Results:**

Response rate was 15.2% of women/11.2% of men who were sent an invitation email, and 38.4% of women/28.3% of men who opened the email. In the last 12-months, 22.1% of women and 24.0% of men had experienced IPV, while across the adult lifetime, 45.1% of women and 35.0% of men had experienced IPV. These figures are higher than an Australian community sample. Non-partner sexual assault had been experienced by 18.6% of women and 7.1% of men, which was similar to national community sample. IPV survivors were 2-3 times more likely to have experienced physical, sexual or emotional abuse in childhood compared to those without a history of IPV (women OR 2.7, 95% CI 2.4 to 2.9; men OR 2.8, 95% CI 2.0 to 4.1). Since the age of sixteen, 11.7% of men and 1.7% of women had behaved in a way that had made a partner or ex-partner feel afraid of them.

**Conclusions:**

The high prevalence of intimate partner violence and child abuse in this group of nurses, midwives and carers suggests the need for workplace support programs. The findings support the theory that childhood adversity may be related to entering the nursing profession and has implications for the training and support of this group.

## Background

Nurses, midwives and carers (hereafter referred to as ‘nurses’) are frontline responders to patients presenting for healthcare who have experienced domestic and family violence (DFV). DFV can cause fear, loss of control and is associated with a range of harms including social isolation, depression, anxiety, substance abuse and employment disruption [1–3]. This health sequalae of DFV fuels an overrepresentation of survivors attending healthcare services and increasing emphasis placed on nurses and other health professionals to identify and respond to survivor patients [4]. DFV includes behaviour within an intimate or family relationship that causes physical, sexual or psychological harm, including intimate partner violence (IPV), non-stranger sexual assault, family violence and child abuse [5]. When violence against nurses has been the focus of past research, it has usually been in the context of abuse by patients, a major problem in the highly gendered environment of healthcare workplaces [6, 7]. However, adding to this load for nurses is evidence that they may themselves experience a higher prevalence of DFV in their own homes compared to the general community [8–10].

### Community prevalence

International data across more than 150 countries indicates that 27% of all ever-married/partnered women have experienced physical partner violence and/or sexual violence by a partner or somebody else; 13% in the last 12-months [11]. For context to this study setting, in Australia, the lifetime prevalence of physical/sexual IPV is 17% of women and 6% of men; emotional IPV is 25% of women and 17% of men [12]. The 12-month prevalence of physical/sexual IPV is 2.3% of women and 1.1% of men, while 4.8% of women and 4.2% of men have experienced emotional IPV [13]. The gendered nature of DFV in the community is underpinned by the still-evident power disparity between men and women and enduring ideology associating masculinity with dominance [14, 15].

### DFV against nurses

An extensive search of the academic literature (1991-2021) using the search (and associate) terms – ‘intimate partner violence’; ‘domestic violence’; ‘family violence’; ‘nurses and health professionals’ – identified 19 quantitative studies about DFV against nurses globally [8, 9, 16–32]. Originating from 16 countries, most of these studies report DFV prevalence against women nurses, with five studies including men nurses [8, 18, 21, 24, 29]. Of two studies that separate the experience of IPV during the last 12-month by gender, men nurses reported a higher prevalence of combined IPV (between 9 and 16%) than women nurses (between 8.2 and 13.9%), which was not consistent with local community statistics [8, 24]. In the three studies of adult lifetime IPV however, women reported a higher combined IPV prevalence (between 34 and 51%) than men (between 3 and 21%) [18, 24, 29].

Across the five studies of IPV against women nurses in the last 12-months [8, 9, 17, 25, 27], the prevalence of physical or sexual / psychological IPV was 4.5% / 12.0% respectively (nurses and healthcare assistants in the UK) [8] and 35.0% / 48.3% (nurses in India) [27]. Across 18 studies of a combined 8926 women’s experience of IPV, adult lifetime prevalence ranged between from 12.0% (190 nurses in Sweden) [31] to 97.7% (350 nurses and doctors in Pakistan) [25] [9, 16–32]. Across all the previous studies, the prevalence of DFV against nurses has most commonly reflected broader community DFV prevalence in the places where the research was conducted [16, 17, 19, 22, 25, 27, 30]. However, where a difference between DFV against nurse participants and the general community was detected, it was more likely that nurses reported a higher prevalence of DFV [8, 9, 18, 23, 24] compared to the general community. Only two studies have investigated the perpetration of violence against a partner by nurses [21, 24], and only one separated their results by gender, finding that 16.0% of 45 men and 21.4% of 294 women reported that they had ever perpetrated physical IPV [24].

These studies were not without limitations and these included: small sample sizes (*n* < 100) [16, 26, 27, 31, 32]; long recall period (> 12-months) [16, 18–23, 26, 28–32]; lack of use of a validated IPV measure [17, 21, 22, 31]; low (< 10%) or unpublished response rate [8, 18, 21, 25, 27, 28, 32], and results not separated by gender [21]. Only one study has been conducted in Australia and utilised a small sample of 471 women health professionals [9].

The objective of this study was to conduct a larger survey about women and men nurses’ exposure to 12-month and adult lifetime IPV to understand the breadth of abusive experiences in nurses’ lives using similar methodology to the authors previous research with a smaller sample of Australian health professional women (Blinded). Secondary objectives were to investigate, experiences of sexual assault, reproductive coercion, technology-facilitated abuse and child abuse and the prevalence of IPV perpetration.

## Methods

A descriptive, cross-sectional online survey about abuse, health, employment, service use and needs was developed using standardised measures (Table 1). The final survey consisted of 78 questions, with piloting leading to modifications of the wording. Within the project advertisements, pre-reading information and introduction to the survey, participants were advised that the survey focused on health, relationships, experience of violence, work and community; the survey was not labelled a ‘DFV survey’. The decision not to label the survey as focussed on DFV was based on: the safety of participants (i.e. to minimise the risk that an abusive partner monitoring a participant’s emails would become aware of the project); minimising the risk of response bias (i.e. the likelihood that DFV survivors may be more motivated to participate out of a desire to share their experience, or less motivated to participate to avoid trauma reminders); the variety of topics under investigation, and the problem that ‘DFV’ and like terms are interpreted differently by people in the community. The survey introduction detailed information about the survey structure, items, length, authors and local specialist support services. This paper focuses on the prevalence of IPV (including perpetration), sexual assault, technology-facilitated abuse, reproductive coercion, IPV in the workplace, physical/sexual child abuse and exposure to violence between parents when growing up. Women and men’s results are presented separately due to men comprising less than a tenth of all participants as well as the gendered nature of DFV in the community which suggests the possibility of a different pattern of abuse for men [40].

**Table 1:** Survey measures

Variables	Source measure	Number of survey items
Demographics	Australian Bureau of Statistics Personal Safety Survey (ABS PSS) [13]	12
Physical & emotional health	SF-12 [33]	12
Depression	PHQ-4 [34]	2
Anxiety	PHQ-4 [34]	2
Posttraumatic stress disorder	Short Screening Scale for DSM-IV Posttraumatic Stress Disorder [35]	7
Hazardous alcohol consumption	FAST [36]	4
12-month & adult lifetime IPV	CAS [37]	36
12-month & adult lifetime IPV perpetration	Bespoke	5
Physical & sexual child abuse	ABS PSS [13]	4
Witnessing FV	WAV Project [9]	1
Non-partner sexual assault	ABS PSS [13]	5
Digital abuse	TAR Scale [38]	2
Reproductive coercion	Bespoke	2
Workplace impacts of IPV	DV and the Canadian Workplace Survey [2]	11
Health & specialist service use	Bespoke	20
Resiliency	CD-RISC2 [39]	2
Advocacy & support	Bespoke (open-ended questions)	5

### Participants

All current members of the Australian Nursing and Midwifery Federation (ANMF Vic Branch) were eligible to participate. The ANMF (Vic Branch) is an industrial Union and members comprise registered nurses, midwives or carers working in Victoria, Australia. A survey invitation email was sent to all 70,124 women and 6935 men members of the ANMF (Vic Branch). Sent by the ANMF (Vic Branch) Secretary, 27,759 women and 2745 men members opened the email containing information about the project and an online survey link. The survey, conducted via Qualtrics between 30 August 2019 and 7 February 2020, was confidential and voluntary, and completion implied consent [41]. A reminder text message encouraging participation was sent to all potential participants from the ANMF (Vic Branch) Secretary at two time points during data collection.

### Definitions

IPV was defined as physical, sexual and/or psychological violence, including the threat of such violence, occurring within a current or past adult intimate relationship (lasting longer than 1 month) with a partner/boyfriend/girlfriend/husband/wife since the age of 16. This definition applied to survey items about IPV victimisation and perpetration. The Composite Abuse Scale (CAS) is a 30-item validated self-report measure of abusive behaviours during the last 12-months using a 6-point scale (Not in past 12-months/Once/A few times/Monthly/Weekly/Daily/Almost daily) [37]. A total CAS score derived from scores of all items (30 items, each scored 0-5, allowing a possible total score of 0-150) or the total scores for each subscale. Standard cut off scores were used (Severe Combined Abuse = 1 [8 items], Physical Abuse = 1 [7 items], Emotional Abuse = 3 [11 items], Harassment = 2 [4 items]) [37]. The final step in the scoring process involved categorising participants into one of four 12-month IPV categories:

- SCA: Severe combined abuse (severe physical, sexual and/or emotional violence);
- PA + E: Physical abuse combined with emotional abuse and/or harassment (not including sexual abuse);
- PA alone: Physical abuse (not in combination with sexual or emotional abuse or harassment);
- E + H alone: Emotional abuse and/or harassment (not in combination with physical/sexual abuse).

As per a previous study by the authors, the CAS was adapted to measure adult lifetime IPV since the age of 16 (Blinded). Lifetime IPV was defined as qualifying for 12-month abuse (any of the four categories) or an adult lifetime score on the CAS subscales: SCA (1+) or PA + E (2+).

Applying questions from the ABS Personal Safety Survey (PSS), sexual assault was defined as forced (including attempted) sexual activity (i.e. rape) by anyone since the age of fifteen [13]. The definition of sexual assault excluded unwanted sexual touching and offensive sexual behaviour (i.e. indecent emails, exposure, inappropriate sexual comments) since the age of sixteen [13]. Reproductive coercion was defined as the use of force (including attempted) to become pregnant when this was not wanted or to end a wanted pregnancy since the age of sixteen [42]. Technology-facilitated abuse was defined as monitoring via digital software or the distribution (or threat) of nude images/video without permission [38]. Child abuse was defined as physical harm (slapped, hit, beat, kicked, restrained) or sexual activity before the age of fifteen perpetrated by an adult over the age of eighteen [13] and/or witnessing family violence against a parent [9].

### Analysis

The prevalence of DFV was derived from univariate analyses using frequencies and percentages. Odds ratios, 95% confidence intervals (CI) and *P-*values were employed to assess the likely size of the association between demographic and other variables with categories of abuse. Quantitative data was imported, cleaned and coded using SPSS (version 25) [43] and analysed with STATA (version 15) [44]. Ethics approval for the project was granted by the University of Melbourne Human Research and Ethics Committees (Ethics ID: 1953826).

## Results

There were 10,674 women and 772 men ANMF (Vic Branch) members who completed a survey, achieving response rates of between 15.2% (women)/11.2% (men) (those sent an invitation email) and 38.4% (women)/28.3% (men) (those who opened email). Most participants were women (93.1%), born in Australia (77.5%), median age of 47 years (50.4%) who worked in a public hospital more than 3 days a week (59.9%) (Table 2).

**Table 2:** Demographic characteristics of participants compared to the broader ANMF (Vic Branch) and Australian population

Characteristic	No. (%) of all participants	No. (%) of women participants	No. (%) of men participants	No. (%) of ANMF member population^**a**^	ABS PSS population %^**b**^	Australian population %^**c**^
**Sex**	*N* = 11,465	10,629 (92.7)	772 (6.7)	*N* = 87,076	*N* = 21,242	**
Female	10,629 (92.7)	–	–	79,264 (91.0)	15,589	**
Male	772 (6.7)	–	–	7790 (8.9)	5653	**
Non-binary	13 (0.1)	–	–	**	**	**
Preferred not to say	51 (0.4)	–	–	**	**	**
**Age (years)**	(*n* = 11,321)	(*n* = 10,519)	(*n* = 760)			
< 30	1189 (10.5)	1109 (10.5)	75 (9.9)	16,098 (18.5)	15.3	21.8
30-39	2113 (18.7)	1937 (18.4)	167 (22.0)	23,015 (26.4)	19.3	18.4
40-49	2401 (21.2)	2213 (21.0)	173 (22.8)	17,689 (20.3)	18.3	17.3
50-59	3399 (30.0)	3182 (30.2)	209 (27.5)	17,595 (20.2)	17.2	16.2
60-69	2101 (18.6)	1962 (18.7)	134 (17.6)	10,007 (11.5)	16.2	13.5
≥ 70	118 (1.0)	116 (1.2)	2 (0.2)	918 (1.1)	13.6	12.7
**Country of birth**	(*n* = 8831)	(*n* = 8227)	(*n* = 564)			
Australia	6795 (76.9)	6380 (77.5)	384 (68.1)	**	70.5	66.9
English first language	7789 (88.5)	7300 (89.0)	452 (80.6)	**	**	**
**Aboriginal/Torres Strait Islander**	107 (1.2)	104 (1.3)	3 (0.5)	**	**	**
**Intimate relationship status**	(*n* = 10,195)	(*n* = 9497)	(*n* = 649)			
In a current relationship^d^	7361 (72.2)	6827 (71.9)	504 (77.6)	**	57.1	62.4
Relationship during past 12mths^d^	7779 (76.3)	7201 (75.8)	545 (84.0)	**	**	**
Ever been in a relationship	9682 (95.0)	9021 (95.0)	614 (94.6)	**	81.7	78.7
**Sex of current partner**	(*n* = 6776)	(*n* = 6297)	(*n* = 452)	**	**	**
Male	6209 (91.6)	6135 (97.4)	59 (13.1)	**	Women respondents: 62.4 Male respondents: 1.3	Women: 59.8 Men: 0.9
Female	559 (8.2)	158 (2.5)	392 (86.7)	**	Female respondents: 0.3 Male respondents: 40.3	Women: 0.4 Men: 63.8
Non-binary	8 (0.0)	4 (0.1)	1 (0.2)	**	**	**
**Current living situation**						
Living with partner (incl. married)	6111 (69.5)	5673 (69.5)	413 (69.3)	**	47.9	52.5
In a relationship, but not living with partner	556 (6.3)	514 (6.3)	39 (7.0)	**	**	**
Separated	324 (3.7)	306 (3.7)	14 (2.5)	n.a.	4.9	3.1
Divorced	669 (7.6)	634 (7.7)	32 (5.7)	n.a.	12.5	8.4
Widowed	214 (2.4)	209 (2.6)	4 (.7)	**	6.6	4.5
Not in a relationship/single	1179 (13.4)	1093 (13.3)	80 (14.3)	**	42.9	37.6
**Children**	(*n* = 8772)	(*n* = 8177)	(*n* = 558)			
No children	2320 (26.4)	2093 (25.6)	213 (38.2)	**	**	**
Currently pregnant	–	126 (1.1)	–	**	**	**
1+ children living at home	4799 (54.7)	4519 (55.2)	266 (47.7)	**	32.2	34.2
**Sector of work**	(*n* = 8584)	(*n* = 7995)	(*n* = 552)	(*N =* 87,076)	**	**
Public Acute	3947 (46.0)	3678 (46.0)	255 (46.2)	41,280 (47.4)	**	**
Private Acute	824 (9.6)	788 (10.0)	32 (5.8)	10,490 (12.0)	**	**
Public Mental Health	320 (3.7)	244 (3.0)	73 (13.2)	2379 (2.7)	**	**
Private Mental Health	81 (0.9)	66 (0.8)	15 (2.7)	528 (0.0)	**	**
Public Aged Care	801 (9.3)	747 (9.3)	51 (9.2)	1686 (1.9)	**	**
Private Aged Care	724 (8.4)	672 (8.4)	48 (8.7)	12,371 (14.2)	**	**
Other	1887 (22.0)	1880 (22.5)	78 (14.1)	18,342 (21.1)	**	**
**Work hours p/wk**	*(n* = 8596)	*(n* = 8004)	*(n* = 553)			
1-3 days (24 hrs or less)	2138 (24.9)	2072 (25.9)	54 (9.8)	28.0	14.5	12.0
> 3-4 days (25-34 hrs)	3526 (41.1)	3318 (41.4)	193 (34.9)	64.0	9.1	7.1
5+ days (35-44 hrs)	2468 (28.7)	2210 (27.7)	249 (45.0)		39.6	44.5
6+ days (45 hrs+)	464 (5.4)	404 (5.0)	57 (10.2)			

Denominators vary due to missing responses; base = all survey participants who responded

^**^Comparable data either not collected or available

^a^ANMF (Vic Branch) October 2019 data. N.b: ANMF (Vic Branch) population to whom survey information was sent in August 2019 was 77,059 members

^b^2016 PSS data provided by ABS (courtesy of Anthea Saflekos) 16 February 2021

^c^Population data is weighted proportions from PSS, provided by ABS (Anthea Saflekos) 16 February 2021

^d^513 participants were omitted as they had never been in a relationship

Most participants were living with a male partner (98.8%) and two children (31.2%) at the time of the survey. The majority of participants identified they were in a relationship with an opposite-sex partner, although around a quarter (27.3% men / 22.9% women) chose not to disclose the sex of their partner and more than one in ten men (13.1%) and 2.5% of women were in a same-sex relationship.

### Adult lifetime prevalence of intimate partner violence

**Fig. 1:**
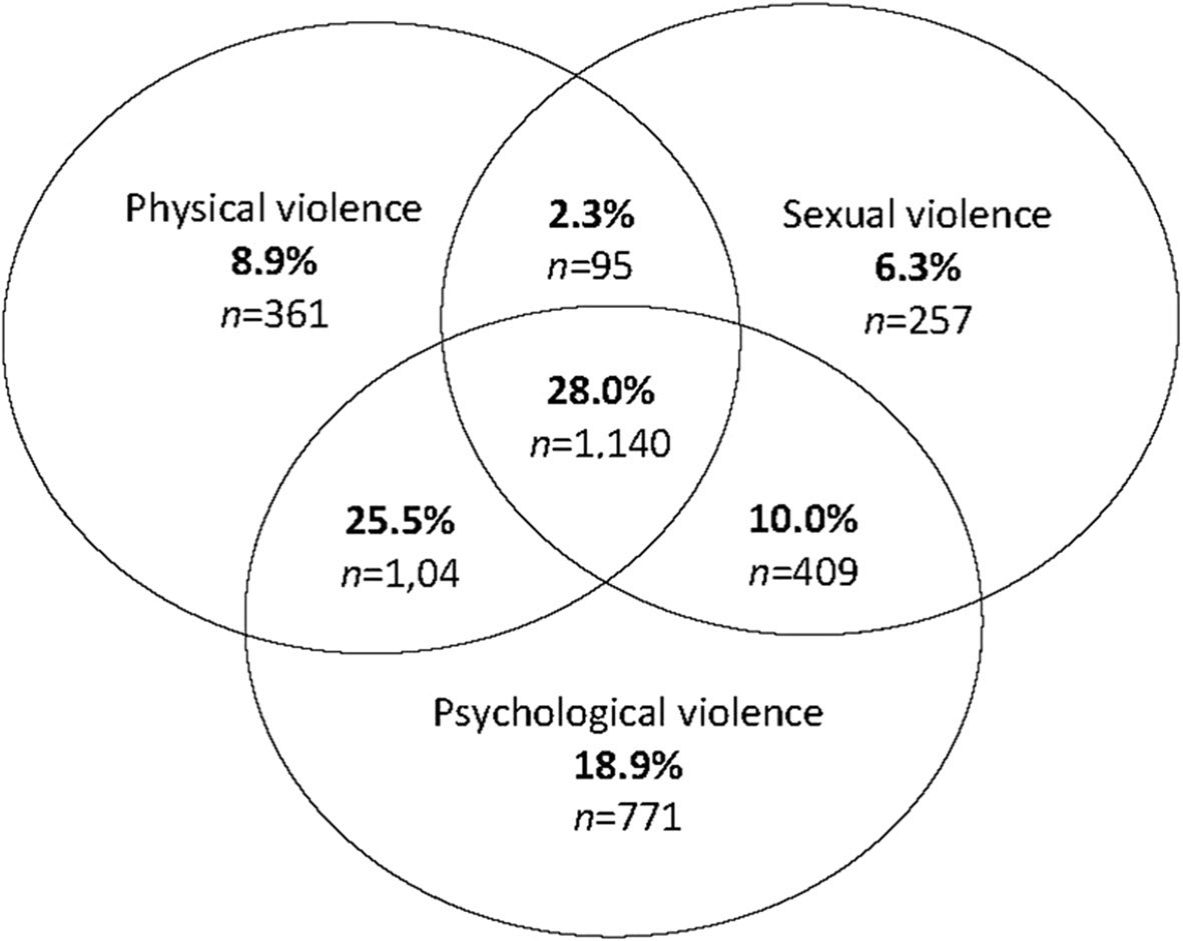
Women’s overlap of physical, sexual and psychological IPV since sixteen years old (*n* = 4074)

**Fig. 2:**
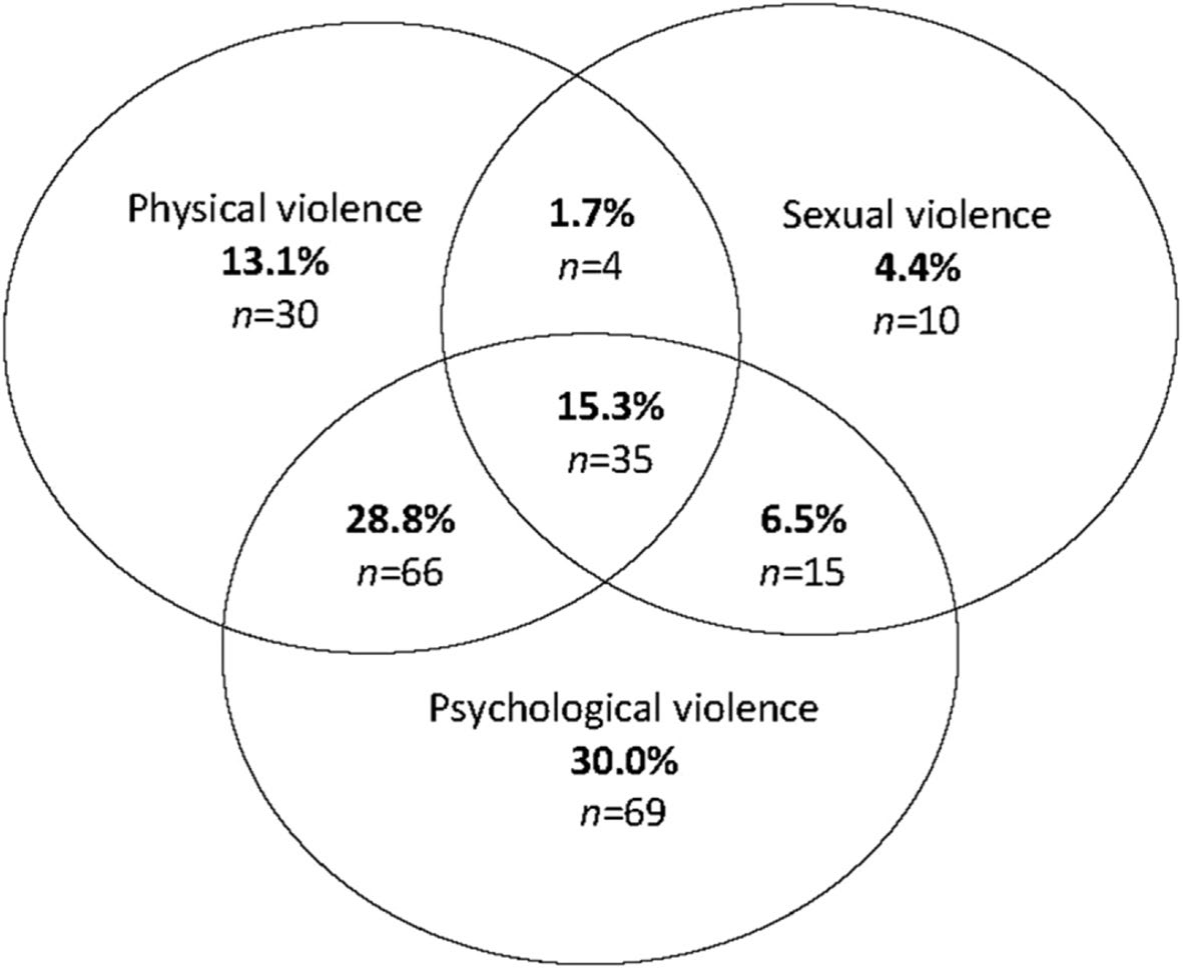
Men’s overlap of physical, sexual and psychological IPV since sixteen years old (*n* = 229)

Nearly half of women participants (45%) and a third of male participants (35.0%) had experienced physical, sexual and emotional abuse in at least one intimate relationship since the age of sixteen (Table 3). Almost double the proportion of women participants (32.2%) than men (17.2%) reported having ever felt afraid of a partner or ex-partner. For 1 in 10 of these women (10.1%) and 1 in 4 men (25.1%), their abusive partner was also a work colleague and worked with them at the time of the abuse. Figures 1 and 2 are diagrams of women’s and men’s experience of lifetime IPV by violence type. Among survivor women, the largest group (28.0%) had lifetime experiences of all three types of IPV: physical, sexual and psychological; for survivor men, it was psychological violence and harassment (30.0%). Most survivor women (81.1%) reported exposure to physical and/or sexual violence by a partner in combination with psychological violence. Physical and/or sexual violence alone was less prevalent for both women (17.5%) and men (19.2%). The prevalence of lifetime IPV was the same for Aboriginal and/or Torres Strait Islander women as other women participants (44.4%), however, women born overseas were under-represented among lifetime IPV survivors (38.7% compared to 45.9%).

**Table 3:** Adult lifetime and 12-month prevalence of intimate partner violence ^a.^ Values are numbers (percentages)

Intimate partner violence	Women’s lifetime prevalence	Men’s lifetime prevalence	Women’s 12-month prevalence	Men’s 12-month prevalence
	*n* = 8982	*n* = 611	*n* = 8982	*n* = 611
Fear of partner ^a^	2894 (32.2)	105 (17.2)	552 (7.7)	33 (6.1)
IPV Category (CAS)	*n* = 8760	*n* = 595	*n* = 7847	*n* = 570
Severe physical, emotional and/or sexual combined abuse	2315 (26.4)	95 (16.1)	373 (4.8)	29 (5.1)
Physical abuse and emotional/harassment	739 (8.4)	45 (7.6)	207 (2.6)	15 (2.6)
Emotional abuse and/or harassment alone	837 (10.0)	77 (12.9)	700 (8.9)	65 (11.4)
Physical abuse alone	328 (3.7)	24 (4.1)	96 (1.2)	9 (1.6)
Sexual assault (rape) by partner	1925 (22.2)	65 (11.1)	309 (3.5)	17 (2.8)
**Total: Fear of partner and/or abuse**	4055 (45.1)	214 (35.0)	1540 (22.1)	127 (24.0)

Denominators vary due to missing responses; base = all survey participants who responded

^a^476 women & 35 men participants omitted because they had never been in a relationship

A minority of women participants (5.8%) had experienced behaviour by a partner that interfered with their capacity to make independent decisions about their reproductive health. Some had been forced to become pregnant when they did not want to be (1.7%) and others had been forced to terminate a wanted pregnancy (4.6%) (Table 4). A similar proportion of women (5.4%) and men (5.2%) reported their partner had used technology to facilitate abuse to track their whereabouts or share nude images/video of them without their consent (Table 4).

**Table 4:** Technology-facilitated abuse and reproductive coercion prevalence. Values are numbers (percentages)

Types of abuse	Women’s lifetime prevalence	Men’s lifetime prevalence
	*n* = 8746	*n* = 595
**Technology-facilitated abuse**	468 (5.4)	31 (5.2)
Tracked me without consent	262 (3.0)	22 (3.7)
Distributed images/video without consent	258 (2.9)	15 (2.5)
	*n* = 8294	N/A
**Reproductive coercion**	485 (5.8)	N/A
Forced to become pregnant	140 (1.7)	N/A
Forced to end pregnancy	384 (4.6)	N/A

Denominators vary due to missing responses; base = all survey participants who responded

*N/A* Women-only item

### Twelve-month prevalence of intimate partner violence

**Fig. 3:**
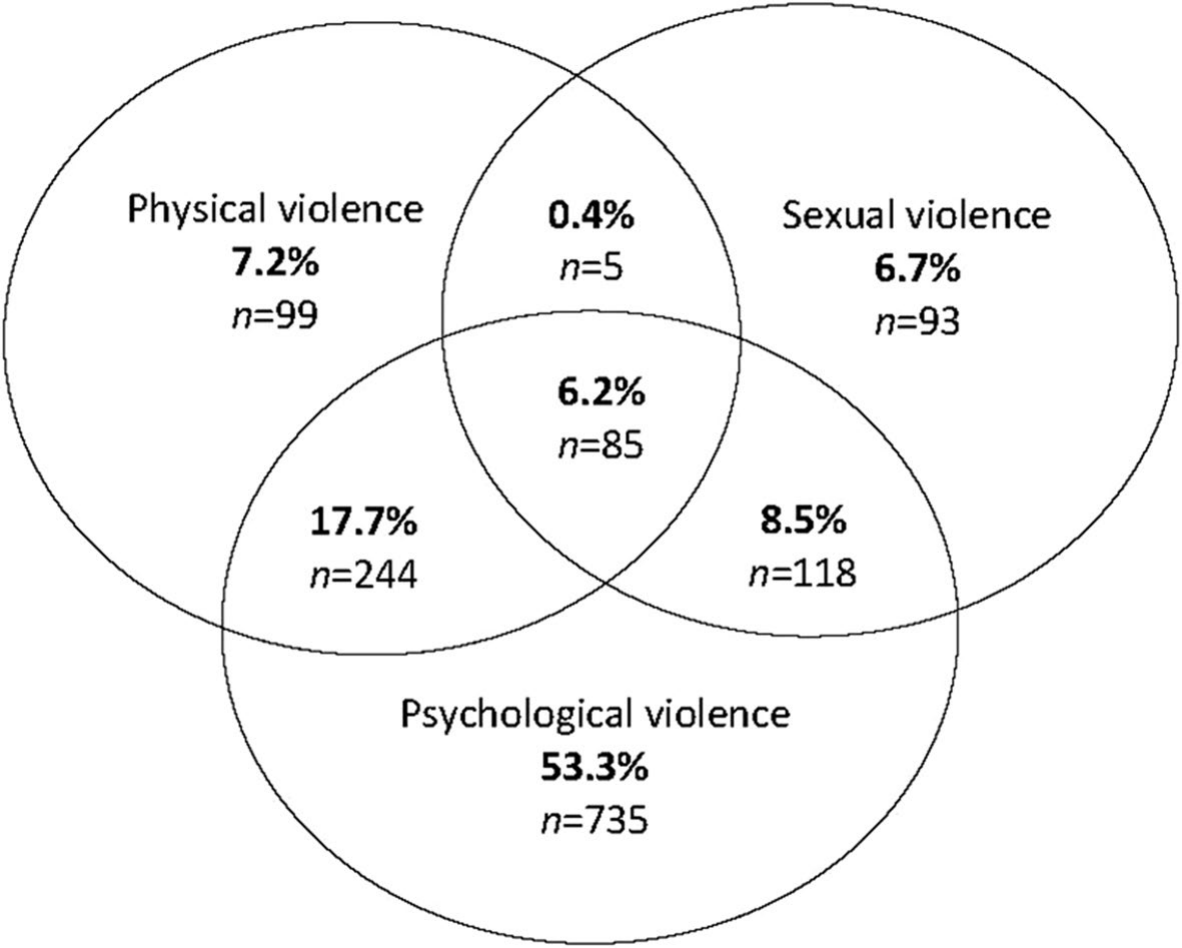
Women’s overlap of physical, sexual and psychological IPV in the last 12-months (*n* = 1379). *Insufficient data to report corresponding survivor men’s data

The 12-month IPV prevalence among women was 22.1%, while for men it was 24.0%. This represented a third of women and half of men who had experienced adult lifetime IPV over a period longer than the last year. Among survivor women, the frequency of abusive acts against them was greater, more likely to be sexually abusive and to cause fear, when compared with survivor men (Table 2, Fig. 3). More than half (54.1%) of survivor women and close to half of survivor men (43.3%) had children at home who may have been exposed to the violence.

### Sexual assault (non-partner) during adulthood

**Fig. 4:**
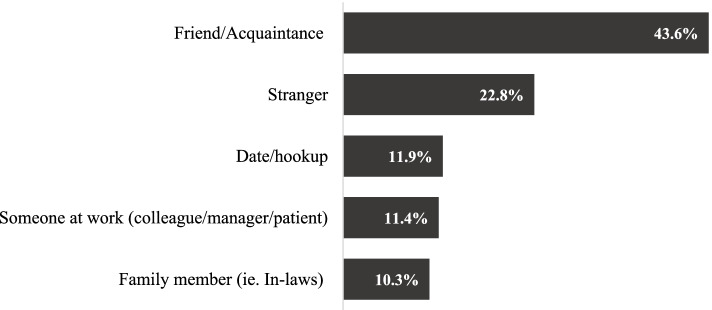
Perpetrator of women’s (non-partner) sexual assault

Sexual assault perpetrated by somebody other than an intimate partner had been experienced by 18.6% of women and 7.1% men (Table 5). In most cases, the offenders of that violence were male friends/acquaintances (43.6%), followed by strangers (22.8%) (Fig. 4). For the one in ten women sexually assaulted by someone with whom they worked, the offender was most frequently described as a work colleague (47.7%), followed by a manager/senior colleague (36.4%) or patient/client (15.9%). For nearly half of survivor women and men, sexual assault was something they had experienced more than once. There was some overlap between women who had been sexually assaulted by someone outside the home as well as by an intimate partner; the odds that a woman had experienced sexual assault by someone other than a partner during adulthood were 3.6 times greater if the woman had survived sexual assault by an intimate partner (95% CI 3.2-4.1).

**Table 5:** Adult sexual assault prevalence. Values are numbers (percentages) unless otherwise stated

Types of abuse	Women’s lifetime prevalence	Men’s lifetime prevalence
	*n* = 8696	*n* = 593
**Sexual assault** (since 15 yrs)	1618 (18.6)	42 (7.1)
Male perpetrator	1589 (99.5)	31 (75.6)
Perpetrator relationship	*n* = 1320	*n* = 35
Stranger	302 (22.8)	*
Friend/acquaintance	580 (43.9)	*
Date/hook-up	157 (11.9)	*
Patient	24 (1.8)	*
Colleague	72 (5.4)	*
Manager/senior	55 (4.2)	*
Someone in the family (i.e. in-laws)	130 (9.8)	*
	*n* = 8696	*n* = 593
Perpetrator worked at same workplace	146 (9.6)	*
More than 1 incident of sexual assault	679 (42.0)	23 (54.8)

Denominators vary due to missing responses; base = all survey participants who responded

*Numerator too small to report (≤15)

### Child abuse

Physical or sexual abuse in childhood had affected a third of women participants and nearly half of men (Table 6). Sexual child abuse was more prevalent against women as girls and most likely to be perpetrated by somebody within the family; men as boys were more commonly exposed to physical abuse, also usually perpetrated by someone within the family. The predicted odds of reporting IPV in adulthood were 2-3 times greater if the survivor had experienced child abuse (women OR 2.7, 95% CI 2.4 to 2.9; men OR 2.8, 95% CI 2.0 to 4.1).

**Table 6:** Child abuse ^a^. Values are numbers (percentages) unless otherwise stated

Child abuse type	Women as girls prevalence	Men as boys prevalence
	*n* = 8652	*n* = 589
Physical child abuse	2487 (28.7)	231 (39.2)
	(*n* = 2261)	(*n* = 210)
Within the family ^b^	2126 (94.0)	175 (83.3)
Outside the family ^c^	135 (6.0)	35 (16.7)
Sexual child abuse	1223 (14.1)	67 (11.4)
	(*n* = 1103)	(*n* = 59)
Within the family	593 (53.8)	21 (35.6)
Outside the family	510 (46.2)	38 (64.4)
	(*n* = 8637)	(*n* = 588)
FV between parents while growing up	2150 (24.9)	156 (26.5)
**Total** (physical/sexual abuse and/or FV exposure)	**3797 (44.0)**	**298 (50.7)**

Denominators vary due to missing responses; base = all survey participants who responded

^a^Abusive behaviour before the age of 15 years by someone aged 18 plus years

^b^Examples of physical & sexual abuse perpetrators within the family are father, uncle, cousin

^c^Examples of physical & sexual abuse perpetrators outside the family are teacher, neighbour, family friend

### IPV perpetration

Both men and women participants who had acted abusively against a partner in the last 12-months were more likely than not to also identify as the victim of partner abuse IPV during that time. For example, 8.0% of men disclosed the use of controlling, threatening or physically/sexually abusive behaviour against their partner in the last year, and 55.0% of them had also been the victim of IPV. Among women, 6.0% had acted controlling, threatening or physically/sexually abusive towards a partner in the last year, with 57.6% having been the victim of IPV during the same period. Since the age of 16 years, 17.6% of men and 16.0% of women had acted abusively towards a partner; for 60.6% of those men and 78.1% of women, they had also been a victim/survivor during that time. A gendered difference was reported on the measure of causing a partner to feel afraid: across the adult lifetime, 11.7% men and 1.7% of women reported causing their partner to feel fearful.

## Discussion

### DFV prevalence in the context of the literature

The findings of this study indicate that nurses, midwives and carers may be overrepresented as victim/survivors of DFV (12-month and adult lifetime IPV, adult sexual assault and child abuse) compared to the general community among whom they live and provide care. While community studies assess IPV differently to this study – the community measure (Australian Bureau of Statistics Personal Safety Survey [13]) defines an intimate partner in narrower terms (either ‘co-habiting’ or ‘boyfriend/date’) and assesses IPV using fewer items, for example – the overrepresentation of IPV reported by the nursing men and women in this study compared to the general Australian community is stark. Between 22 and 24% of women and men nurses in this study reported IPV in the last 12-months, with 7.7% of women characterising that as inducing fear. This represents an increased rate of women’s physical/sexual IPV up to 4.5 times higher than the general Australian community, with emotional abuse up to 2 times higher. Among men, the difference in reported 12-month physical/sexual IPV was up to 10 times higher, while emotional abuse was up to 3 times higher than reported by men in the community [13]. The prevalence of adult lifetime IPV (since 16 years) was also overrepresented in our sample compared to the community: physical/sexual IPV among women participants was more than double that reported by women in the community, while for men participants the increase was greater than 5-fold [13]. Further, women and men in our sample reported adult lifetime emotional IPV up to twice the rate of women and men in the community [45].

The overrepresentation of adult lifetime IPV survivors is important to place within the childhood context of abuse. Childhood abuse (physical/sexual or IPV between parents) was reported by men participants at four times the rate of the general male community and 2.2 times more commonly by women participants [12]. A history of child abuse was associated with up to 3 times increased odds of reporting IPV in adulthood, especially for men participants. While a life course affect could not be directly (causally) investigated in this study, it is indicated by these findings. This result is consistent with the is a wealth of evidence linking adverse circumstances in early life to an increased risk of violence exposure, among other harms, in later life [12, 40].

### Male participants

The comparable IPV prevalence between men and women participants in the previous 12-months was surprising given the substantial divergence from community statistics that this represents [13]. However, the finding is consistent with the two other studies of male nurses within the same timeframe [8, 24]. Similarly, the finding of lower adult lifetime IPV prevalence among men compared to women was aligned with the three other studies of this topic [18, 24, 29]. Reflecting the gendered nature of nursing, the present study and the other background studies have included a much smaller proportion of men to women participants. The men in this study may not be representative of men in broader Australian society; for example, the incidence of same-sex partnerships was more than 10 times higher in the present sample compared to the community average [13]. Men in nursing may not reflect traditionally narrow representations of masculinity [14] and may have been more willing to participate at least in part because the survey gave them a voice for their experience that they otherwise do not have [46].

### Wounded healers

This study adds to the wounded healer evidence-base that has previously uncovered an association between childhood adversity in the backgrounds of social workers, mental health clinicians and counsellors compared to the general community [47, 48]. The findings of the present study suggest that nurses, midwives and carers may have a higher prevalence than others of adversity and abuse, consistent with the findings of several international studies of DFV against nurses [8, 9, 18, 23, 24]. Could DFV be related to nurses’ choice of profession, perhaps because of an enhanced motivation and/or capacity to support others born of experience? [49, 50]. Or, perhaps nurses are more willing than others to identify and disclose DFV to researchers because they are aware of the commonality and manifestations of DFV gleaned from practice [9]. Could survivor nurses face additional barriers to accessing services because of extra concerns about their privacy or increased self-stigma and shame from a perception that those whose job involves supporting survivor patients should not experience DFV themselves? [10]. Whatever the factors associating nursing with an increased prevalence of childhood and adult familial trauma, many of the participants in this study indicated they carry a sizable trauma load, which is likely to get added to in the course of routine patient care [51].

### Vicarious trauma

All nurses, given time, are likely to be exposed to vicarious trauma in their role responding to patients during times of distress and crisis [52, 53]. Stemming from empathetic engagement, vicarious trauma is a transformation of the inner world of the those who listen to, observe and intervene in the trauma of others and can resemble signs of post-traumatic stress [52, 54]. Research suggests that upon a background of personal (primary) trauma, a vicarious (secondary) trauma response may take affect earlier and more severely than among those without a personal history of trauma [48, 50]. This warns of a potentially heavy cumulative trauma load for survivor nurses [9], especially when the patient aggression and harassment that nurses are routinely exposed to is added [7]. It is likely that vicarious and cumulative trauma will be at play for different nurses at different times, so healthcare workplaces and the unions and services that support nurses must be prepared and responsive.

### Strengths and limitations

Strengths of this study include the sample size establishing it as the largest study of IPV among women and men health professionals to date. The study utilised a comprehensive measure of IPV [37], reporting the prevalence of previously understudied forms of interpersonal abuse among nurses, including reproductive coercion, technology-facilitated abuse [38] and use of abusive behaviours. The response rates of 14.9 and 37.6% present a limitation, albeit a difficult one to avoid given the survey’s sensitivity and length and the work/life demands of this population. Further, the response rate is comparable or higher than at least half of the studies in this field [8, 18, 21, 25–28, 32]. The margin of error across all the men’s data was 4% (women’s data < 1%) and due to missing responses, the relative standard error of men’s IPV prevalence data was 12% (women’s data 4%), indicating interpretive caution is required. While overreporting is regarded as rare in DFV research, survivors may have been more interested or willing to take part in this study than others and if respondents differed from non-respondents in meaningful ways, this may have affected the study’s conclusions [55, 56].

### Implications

The present research exposing the high familial trauma load among a large sample of nurses, midwives and carers has implications for healthcare workplace culture, managers and colleagues. Listening to nurses with a lived experience of DFV, encouraging them to be part of any change, is central to workplaces getting the survivor response right [57, 58]. For healthcare organisations to be responsive to the needs of people who have experienced DFV, a trauma and violence-informed approach must be prioritised [59]. This includes infusing all levels of the workplace with an understanding of the commonality of primary and secondary trauma and its centralising role in some survivors’ lives. Working to create and maintain the conditions that enable both patients *and* staff to feel a sense of control, choice and power within the healthcare system. If managers and staff know how to recognise DFV indicators and can respond to staff disclosures in a sensitive and evidence-based way, then survivors should receive information about onsite and offsite support, offered roster flexibility, ensured confidentiality and given control over next steps [60, 61]. Since colleagues may be the first person to whom a disclosure is made, they need to know where to access additional information and support they might need [60, 62]. Organisations have a significant stake in both mitigating against the impacts of vicarious trauma and strengthening staff resilience. Leadership and resource provision is needed to enable staff to practice self and family care, task variation, research, supervision and other evidence-based workplace strategies that can improve psychological wellbeing and reduce work stress [63]. Further, given the proportion of DFV survivor staff in healthcare workplaces that are indicated by these findings, these workplaces have a responsibility to ensure DFV-specific staff support is available and to evaluate the take up, effectiveness and cost of such support [64].

## Conclusion

This study, the largest of DFV against health professionals to date, adds to the existing evidence that those at the frontline of healthcare may carry a substantial DFV trauma burden, encompassing violence in their homes during childhood and into their adult relationships. This must be considered in healthcare workplace support provision, training for colleagues, managers and others to whom survivor staff may disclose, and when resourcing health professionals to respond to their patients after DFV. Strengthening DFV support and advocacy for survivor nurses, midwives and carers may, in turn, reinforce their frontline work responding to the health needs and recovery journeys of patients.

## Data Availability

The dataset supporting the conclusions of this article can be provided as an additional file. At present, the data and materials (survey) are not publicly available but can be obtained from the authors upon reasonable request (please contact: elizabeth.mclindon@unimelb.edu.au). The Composite Abuse Scale and many of the other measures in Table 1 are publicly available [2, 13, 33–39].

## Abbreviations

CAS: Composite Abuse Scale
CI: Confidence interval
DFV: Domestic and family violence
E + H: Emotional abuse/harassment
IPV: Intimate partner violence
OR: Odds ratio
PA: Physical abuse
PA + E: Physical and emotional abuse
PSS: Personal safety survey
SCA: Severe combined abuse

## References

[1] Devries KM, Mak JY, Bacchus LJ, Child JC, Falder G, Petzold M (2013). Intimate partner violence and incident depressive symptoms and suicide attempts: a systematic review of longitudinal studies. PLoS Med.

[2] Wathen CN, MacGregor JCD, MacQuarrie BJ (2016). Relationships among intimate partner violence, work, and health. J Interpers Violence.

[3] World Health Organization (2013). Responding to intimate partner violence and sexual violence against women. WHO clinical and policy guidelines.

[4] García-Moreno C, Hegarty K, D'Oliveira A, Koziol-McLain J, Colombini M, Feder G (2015). The health-systems response to violence against women. Lancet..

[5] Krug E, Dahlberg L, Mercy J, Zwi A, Lozano R (2002). World report on violence and health.

[6] Australian Institute of Health and Welfare Nursing and Midwifery Workforce 2015 ACT, Australia: Australian Institute of Health and Welfare. 2016 Available from: https://www.aihw.gov.au/reports/workforce/nursing-and-midwifery-workforce-2015/contents/who-are-nurses-and-midwives.

[7] Shea T, Sheehan C, Donohue R, Cooper B, De Cieri H (2017). Occupational violence and aggression experienced by nursing and caring professionals. J Nurs Scholarsh.

[8] Cavell Nurses’ Trust (2016). Skint, shaken yet still caring. But who is caring for our nurses?.

[9] McLindon E, Humphreys C, Hegarty K (2018). “It happens to clinicians too”: an Australian prevalence study of intimate partner and family violence against health professionals. BMC Womens Health.

[10] Dheensa S, McLindon E, Spencer C, Pereira S, Shresta S, Emsley E, et al. Healthcare Professionals' own experiences of domestic violence and abuse: a Meta-analysis of prevalence and systematic review of risk markers and consequences. Trauma Violence Abuse. 2022;0(0):1–18. 10.1177/15248380211061771 PMC10240650 34978481

[11] World Health Organization (2021). Violence against women prevalence estimates, 2018: Global, regional and national prevalence estimates for intimate partner violence against women and global and regional prevalence estimates for non-partner sexual violence against women.

[12] Australian Institute of Health and Welfare (2018). Family, domestic and sexual violence in Australia 2018.

[13] Australian Bureau of Statistics (2016). Personal Safety, Australia.

[14] Flood M, Pease B (2009). Factors influencing attitudes to violence against women. Trauma Violence Abuse.

[15] Our Watch & VicHealth (2015). Change the story: a shared framework for the primary prevention of violence against women and their children in Australia.

[16] Al-Natour A, Gillespie G, Wang L, Felblinger D (2014). A comparison of intimate partner violence between Jordanian nurses and Jordanian women. J Forensic Nurs.

[17] Bracken M, Messing J, Campbell JC, La Flair L, Kub J (2010). Intimate partner violence and abuse among female nurses and nursing personnel: prevalence and risk factors. Issues Mental Health Nurs.

[18] Carmona-Torres JM, Recio-Andrade B, Rodríguez-Borrego MA (2017). Intimate partner violence among health professionals: distribution by autonomous communities in Spain. Rev Esc Enferm.

[19] Christofides N, Silo Z (2005). How nurses' experiences of domestic violence influence service provision: study conducted in north-west province, South Africa. Nurs Health Sci.

[20] Diaz-Olavarrieta C, Paz F, de la Cadena C, Campbell JC (2001). Prevalence of intimate partner abuse among nurses and nurses' aides in Mexico. Arch Med Res.

[21] Early M, Williams R (2002). Emergency nurses' experience with violence: does it affect nursing care of battered women?. J Emerg Nurs.

[22] Janssen P, Basso M, Costanzo R (1998). The prevalence of domestic violence among obstetric nurses. Womens Health Issues.

[23] Khan A, Karmaliani R, Saeed Ali T, Asad N, Madhani F (2014). Lifetime prevalence of emotional/psychological abuse among qualified female healthcare providers. Sociol Mind.

[24] Mitchell V, Parekh K, Russ S, Forget N, Wright S (2013). Personal experiences and attitudes towards intimate partner violence in healthcare providers in Guyana. Int Health.

[25] Oliveira AR, D'Oliveira AFPL (2008). Gender-violence against the female nursing staff of a Brazilian hospital in Sao Paulo City. Rev Saude Publica.

[26] Ortlepp K, Nkosi ND (1993). The relationship between spouse abuse and subjective job-related variables in a sample of employed women. S Afr J Psychol Suid-Afrikaanse Tydskrif Vir Sielkunde.

[27] Sharma K, Vatsa M (2011). Domestic violence against nurses by their marital partners: a facility-based study at a tertiary care hospital. Indian J Community Med.

[28] Shokre ES, Ahmed AA (2017). Domestic violence among female nurses: prevalence, effects, and underlying factors. Malaysian J Nurs.

[29] Siltala HP, Holma JM, Hallman M (2019). Family violence and mental health in a sample of Finnish health care professionals: the mediating role of perceived sleep quality. Scand J Caring Sci.

[30] Stenson K, Heimer G (2008). Prevalence of experiences of partner violence among female health staff - relevance to awareness and action when meeting abused women patients. Womens Health Issues.

[31] Sundborg EM, Saleh-Stattin N, Wandell P, Tornkvist L (2012). Nurses' preparedness to care for women exposed to intimate partner violence: a quantitative study in primary health care. BMC Nurs.

[32] Selek S, Vural M, Cakmak I (2012). Abused nurses take no legal steps - a domestic violence study carried out in eastern Turkey. Psychiatr Danub.

[33] Ware JE, Kosinski M, Keller SD (1996). A 12-item short-form health survey - construction of scales and preliminary tests of reliability and validity. Med Care.

[34] Kroenke K, Spitzer RL, Williams JBW, Lowe B (2009). An ultra-brief screening scale for anxiety and depression: the PHQ-4. Psychosomatics..

[35] Breslau N, Peterson EL, Kessler RC, Schultz LR (1999). Short screening scale for DSM-IV posttraumatic stress disorder. Am J Psychiatr.

[36] Hodgson R, Alwyn T, John B, Thom B, Smith A (2002). The Fast alcohol screening test. Alcohol Alcohol.

[37] Hegarty K, Bush R, Sheehan M (2005). The composite abuse scale: further development and assessment of reliability and validity of a multidimensional partner abuse measure in clinical settings. Violence Vict.

[38] Brown C, Hegarty K (2021). Development and validation of the TAR scale: a measure of technology-facilitated abuse in relationships. Comput Hum Behav Rep.

[39] Vaishnavi S, Connor K, Davidson JRT (2007). An abbreviated version of the Connor-Davidson resilience scale (CD-RISC), the CD-RISC2: psychometric properties and applications in psychopharmacological trials. Psychiatry Res.

[40] World Health Organization (2017). Violence Against Women.

[41] Qualtrics (2020). Qualtrics. August 2019 ed.

[42] Tarzia L, Hegarty K (2021). A conceptual re-evaluation of reproductive coercion: centring intent, fear and control. Reprod Health.

[43] IBM Corp (2017). IBM SPSS Statistics for Windows.

[44] StataCorp (2015). Stata Statistical Software: Release 15.

[45] Australian Institute of Health Welfare (2019). Family, Domestic and Sexual Violence in Australia: Continuing The National Story 2019.

[46] Robinson A, Rowlands J (2006). The Dyn project: supporting men experiencing domestic abuse.

[47] Elliott D, Guy J (1993). Mental-health professionals versus non-mental-health professionals - childhood trauma and adult functioning. Prof Psychol Res Pract.

[48] Newcomb M, Burton J, Edwards N, Hazelwood Z (2015). How Jung's concept of the wounded healer can guide learning and teaching in social work and human services. Adv Soc Work Welfare Educ.

[49] Butler LD, Maguin E, Carello J (2018). Retraumatization mediates the effect of adverse childhood experiences on clinical training-related secondary traumatic stress symptoms. J Trauma Dissociation.

[50] Jenkins SR, Mitchell JL, Baird S, Whitfield SR, Meyer HL (2011). The counselor's trauma as counseling motivation: vulnerability or stress inoculation?. J Interpers Violence.

[51] Pihl-Thingvad J, Elklit A, Brandt LLP, Andersen LL (2019). Occupational violence and PTSD-symptoms a prospective study on the indirect effects of violence through time pressure and nontraumatic strains in the occupational context. J Occup Environ Med.

[52] Bell H, Kulkarni S, Dalton L (2003). Organizational prevention of vicarious trauma. Fam Soc.

[53] Sinclair S, Raffin-Bouchal S, Venturato L, Mijovic-Kondejewski J, Smith-MacDonald L (2017). Compassion fatigue: a meta-narrative review of the healthcare literature. Int J Nurs Stud.

[54] McCann IL, Pearlman LA (1990). Vicarious traumatization: a framework for understanding the psychological effects of working with victims. J Trauma Stress.

[55] Ellsberg M, Heise L, Pena R, Agurto S, Winkvist A (2001). Researching domestic violence against women: methodological and ethical considerations. Stud Fam Plan.

[56] McNutt L, Lee R (2000). Intimate partner violence prevalence estimation using telephone surveys: understanding the effect of nonresponse bias. Am J Epidemiol.

[57] Hague G, Mullender A (2006). Who listens? - the voices of domestic violence survivors in service provision in the United Kingdom. Violence Against Women.

[58] Sweeney A, Perot C, Callard F, Adenden V, Mantovani N, Goldsmith L (2019). Out of the silence: towards grassroots and trauma-informed support for people who have experienced sexual violence and abuse. Epidemiol Psychiatr Sci.

[59] Ponic P, Varcoe C, Smutylo T (2016). Trauma (and violence) informed approaches to supporting victims of violence: Policy and Practice Considerations Canada.

[60] McFerran L (2011). National domestic violence and the workplace survey.

[61] Yragui NL, Mankowski ES, Perrin NA, Glass NE (2012). Dimensions of support among abused women in the workplace. Am J Community Psychol.

[62] Rayner-Thomas M, Dixon R, Fanslow J, Tse C (2016). The impact of domestic violence on the workplace. N Z J Employment Relat.

[63] Kulkarni S, Bell H, Hartman JL, Herman-Smith RL (2013). Exploring individual and organizational factors contributing to compassion satisfaction, secondary traumatic stress, and burnout in domestic violence service providers. J Soc Soc Work Res.

[64] McLindon E, Humphreys C, Hegarty K. Hospital responses to employees who have experienced domestic violence: a qualitative study with survivor health professionals and hospital managers. J Gender Based Violence. 2020;5(2):231–47.

